# Organic Control of Dioctahedral and Trioctahedral Clay Formation in an Alkaline Soil System in the Pantanal Wetland of Nhecolândia, Brazil

**DOI:** 10.1371/journal.pone.0159972

**Published:** 2016-07-27

**Authors:** Laurent Barbiero, Gilles Berger, Ary T. Rezende Filho, Jean-François Meunier, Elisângela R. Martins-Silva, Sonia Furian

**Affiliations:** 1 Géosciences Environnement Toulouse (GET), Observatoire de Midi-Pyrénées, Université de Toulouse, CNRS, IRD, 14 Avenue Edouard Belin, F-31400 Toulouse, France; 2 Institut de Recherche en Astrophysique et Planétologie (IRAP), Observatoire de Midi-Pyrénées, Université de Toulouse, CNRS, IRD, 14 Avenue Edouard Belin, F-31400 Toulouse, France; 3 FAENG—Faculdade de Engenharias, Arquitetura e Urbanismo e Geografia, Universidade Federal do Mato-Grosso do Sul, Cidade Universitária s/n, Caixa Postal 549, 79070–900 Campo Grande-MS, Brazil; 4 Laboratoire de Chimie de Coordination (LCC), Université de Toulouse, CNRS, 205 route de Narbonne F-31077, Toulouse, France; 5 Departamento de Ciências Agrárias e Ambientais, Universidade Estadual de Santa Cruz, Salobrinho 45662900, Ilhéus, Brazil; 6 Departamento de Geografia (DG) Universidade de São Paulo, Avenida Pr. Lineu Prestes 338, Cidade Universitária 05508–900, São Paulo, Brazil; Southwest University, CHINA

## Abstract

Recent studies have focused on the formation of authigenic clays in an alkaline soil system surrounding lakes of the Nhecolândia region, Pantanal wetland. The presence of trioctahedral Mg-smectites (stevensite and saponite types), which requires low Al and Fe contents in the soil solution for its formation, contrasts with the neoformation of dioctahedral Fe-mica (glauconite, and Fe-illite), which instead requires solutions relatively enriched in Al and Fe. This study aims to understand the conditions of co-existence of both, Mg-smectite and Fe-mica a common clay association in former or modern alkaline soil systems and sediments. The study was carried out along an alkaline soil catena representative of the region. The soil organization revealed that Mg-smectite occur in top soil close to the lake, whereas Fe-mica dominate in the clay fraction of deeper greenish horizons a few meters apart. We propose here that this spatial distribution is controlled by the lateral transfer of Fe and Al with organic ligands. Alkaline organic rich solutions (DOC up to 738 mg L^-1^) collected in the watertable were centrifuged and filtered through membranes of decreasing pore size (0.45 μm, 0.2 μm, 30 KDa, 10 KDa, 3 KDa) to separate colloidal and dissolved fractions. Fe, Al, Si, Mg and K were analysed for each fraction. Although the filtration had no influence on Si and K contents, almost 90% of Fe (up to 2.3 mg L^-1^) and Al (up to 7 mg L^-1^) are retained at the first cutoff threshold of 0.45μm. The treatment of the same solutions by oxygen peroxide before filtration shows that a large proportion of Fe and Al were bonded to organic colloids in alkaline soil solution at the immediate lake border, allowing Mg-smectite precipitation. The fast mineralization of the organic matter a few meters apart from the lake favors the release of Fe and Al necessary for Fe-mica neoformation. In comparison with chemical and mineralogical characteristics of alkaline environments described in the literature, the study suggests that the co-existence of trioctahedral Mg-smectite and dioctahedral Fe-mica should be regarded as a standard occurrence in alkaline soil systems with organic rich waters.

## Introduction

The high chemical and structural variability of clay minerals is an obstacle to understanding their mechanism of formation, transformation and alteration. This also applies to the determination of the thermodynamic parameters that are required for digital models based on thermo-kinetic laws. In addition, it is difficult to establish clear relations between structure and chemistry for crystal populations of heterogeneous structure having a grain size of a few hundred nanometers. Therefore in soil systems, the presence and spatial distribution of clay minerals are generally interpreted as a function of their thermodynamic stability in aqueous solutions accompanying water percolation through soil profiles and landscape. This thermodynamic vision is sometimes tempered by considering the recycling of certain chemical elements by forest or crops at the top of the soil profiles [1], [2], [3], [4], [5]. Many reactions at low temperature, resulting in mineralogical changes, are not controlled by thermodynamics but kinetics. Clay transformation such as, for example, smectite to illite conversion, requires not only times but also a mineral precursor. Readers can refer to the work of Lanson et al. [6]for a review in sedimentary basin, Hugget and Cuadros [7]for paleosoils, and Andrade et al. [8]for mangrove soils. In addition, vertical and lateral particle translocation within the soil may alter their distribution along soil systems, and clay minerals may end up at depths and places different from those where they formed. Finally, living organisms may modify deeply the conditions from those generated by the inorganic environmental factors.

This view of clay stability in soils also applies to evaporative environments where the solution concentration by evaporation predominates and controls the soil forming processes. Evaporative environments can be classified into two main types according to the sign of the calcite residual alkalinity (RA_calcite_), defined as the difference between alkalinity and the concentration of calcium in solution [9]. These two types are neutral environments (RA_calcite_ <0) and alkaline environments (RA_calcite_> 0). In naturally alkaline environments, the pH is controlled by the carbonate system and increases through evaporative concentration to reach values up to 10 or 11[10]. In this chemical context, neoformation (without a mineral precursor) of non- or low-aluminous trioctahedral smectites from the soil solution has been reported by many authors [11], [12], [13], [14], [15], [16], [17]; [18], [19]). Minerals such as stevensite, sepiolite, and saponite are frequently identified. They require high pH, Al and Fe depleted- and Si rich-solution for their formation that usually control dissolved Mg concentrations [20]. On the other hand, only a few studies have reported authigenic micas in alkaline environments. It has been first mentioned in the 1960s [21], [22], [23] as a transformation of smectitic precursors, usually by wetting and drying [24], [18], [7], while other studies rather suggest a neoformation process from the soil solution at least for illite [25], [26], [27], [28] and glauconite formation [29]. Whatever the process (neoformation or transformation), the formation of Al- and Fe-bearing dioctahedral clay minerals requires a high availability of Si and K, as well as Fe and Al. As these latter elements are generally observed at low concentrations in natural waters and soil solutions, their availability appears as a serious chemical constraint.

In the soil system around alkaline lakes in the Pantanal wetlands, the occurrence of both trioctahedral Mg-smectites and dioctahedral Fe-micas have been mentioned by Furquim et al. [19], [30], [31]. Although they require different chemical composition of the soil solution for their formation, both Mg-smectites and Fe-micas have been identified as authigenic clay minerals by direct precipitation from the soil solution. In this work, we focus on the distribution of these clay minerals in the soil system in relation with the seasonal hydrological functioning of the lakes and with the chemistry of circulating waters, in order to understand the conditions that could explain the co-existence of such di- and trioctahedral clay minerals. In particular, we explore the relationship between the amounts of organic matter, a peculiarity of alkaline environments, and the availability of the less soluble illite constituents, Al and Fe, as a key for the neoformation of illitic material.

### Pantanal of Nhecolândia: the site background

**Fig 1:**
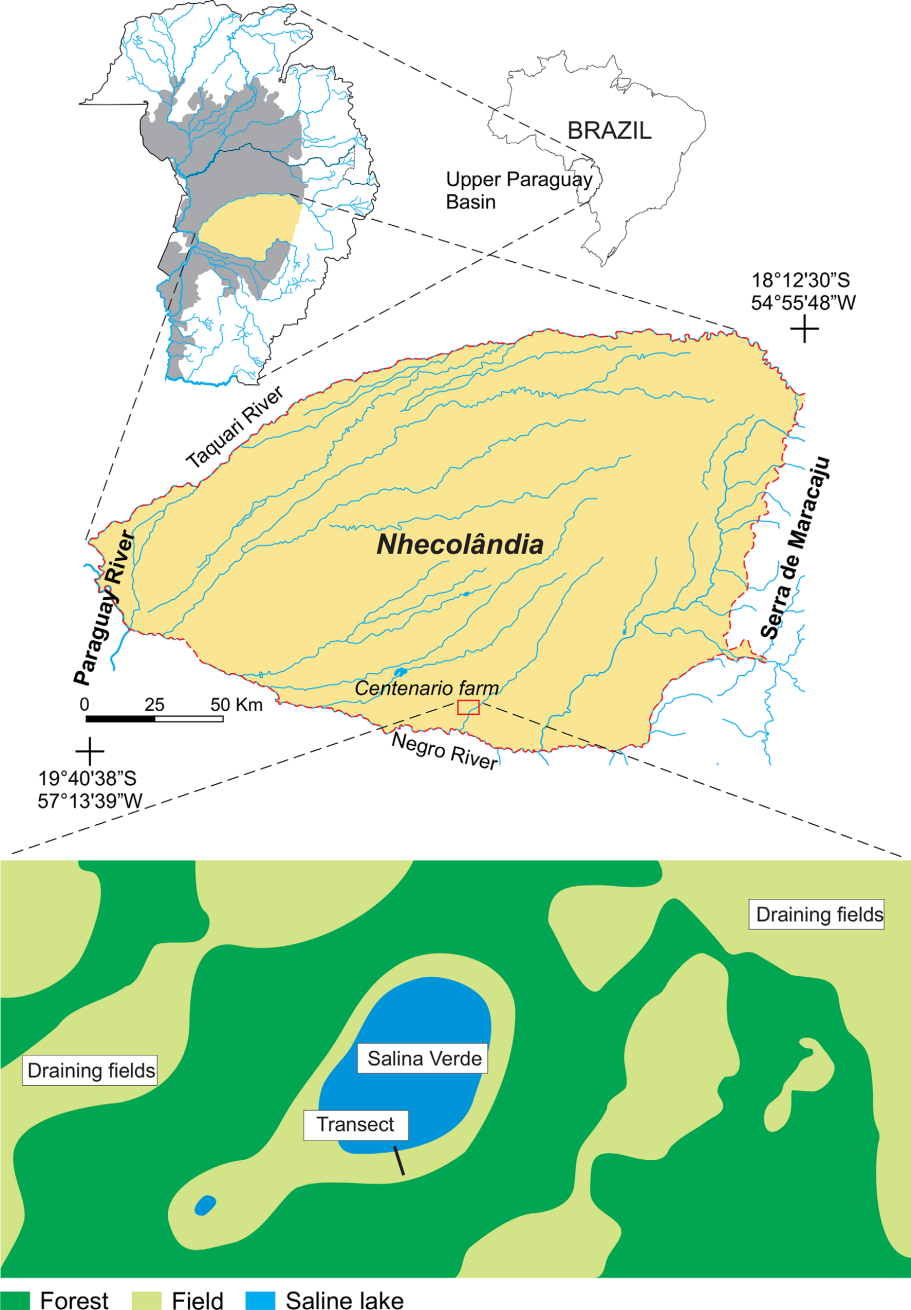
Location of the studied transect at the border of Salina Verde in the Pantanal wetland of Nhecolândia.

In the Brazilian Pantanal wetland, Nhecolândia is a specific sub-region with about 15,000 lakes, including about 500 saline-alkaline ones [32]. It lies on the southern half of the vast Taquari alluvial fan, one of the largest alluvial fans in the world [33], [34]. With a total surface area of about 24,000 km^2^, the region is delimited in the north by the Taquari River, in the south by the Negro River, in the west by a portion of the Paraguay River, and in the east by the Maracajú Plateau, which corresponds to the southeastern edge of the Pantanal wetland (Fig 1). Nhecolândia shows relatively closed drainage with little connection to major fluvial systems. Semihumid climate patterns, classified as tropical humid with short dry season (“Aw” type in the Köppen classification), are controlled by the seasonal migration of the Intertropical Convergence Zone (ITCZ). The mean annual air temperature is ~25°C ranging from 21°C during dry winters to 32°C during rainy summers [35]. The mean annual precipitation (P) is ~1100 mm, and the annual evapotranspiration (ETP) is approximately 1400 mm, providing a hydrological deficit of ~300 mm [36]. At the local level, the strong thermal contrast between saline lakes and surrounding forested areas causes day–night alternating winds that enhance evaporation [37]. Quaternary sediments cover Nhecolândia, arising from sandstone formations that make up the Maracajú Plateau.

**Fig 2:**
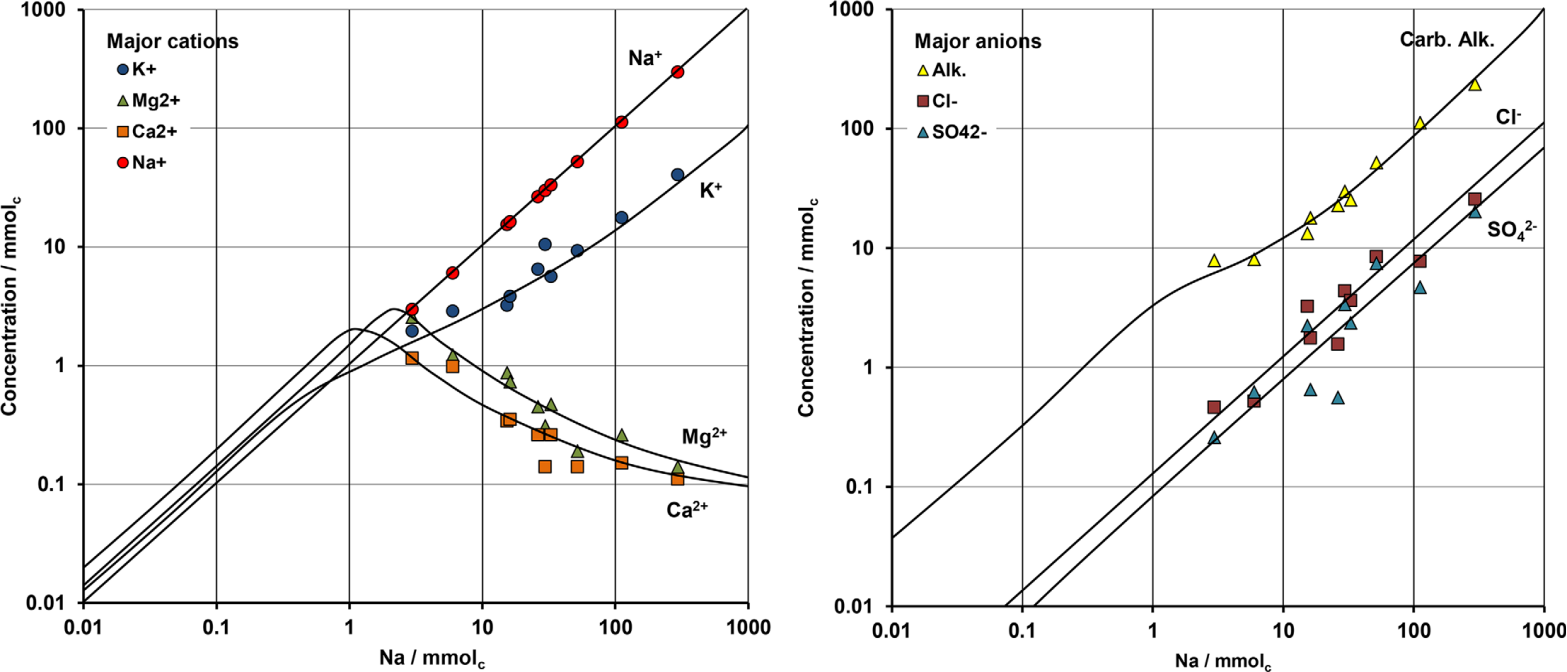
Concentration diagrams based on Na contents showing the regional trends (solid line) compiled from Furian et al. [32] and the chemistry of waters collected in auger holes along the transect of Salina Verde. Note the sigmoidal shape of dissolved K along the concentration process.

The Electrical Conductivity (EC) of surface waters ranges from 0.02 mS cm^-1^ to 80 mS cm^-1^. Furian et al. [32] have studied the water chemistry of 147 lakes in central and southeastern Nhecolândia. The study revealed that the chemistry results from evaporative concentration of fresh waters that originate from the sandstone formation of the upland border of the Pantanal [38], [39]. The evaporation of such water, with positive calcite residual alkalinity, results in alkaline solutions with high pH values, close to or above 9.5. The trends of the different dissolved ions along the evaporation process are summarized in Fig 2. The precipitation of calcite causes a decrease of calcium and consequently the sodication of the soil cation-exchange complex. The incorporation of 3 to 5% of Mg in the calcite [40] together with the neoformation of Mg-smectite close to the lakeshore provoke a decrease of Mg. Finally, although the levels of K increase during evaporation, there is a loss in K, which can logically be attributed to its incorporation in the layers during the formation of Fe-mica [30]. Furian et al. [32] have shown that the range in the salinity level of surface waters is controlled by the hydrological functioning of each lake. The saline lakes are partially isolated from the regional fresh groundwater by soil horizons that limit the flux of fresh water into the saline lakes, thereby preventing their dilution. At the maximum of the flood in the Pantanal, the freshwater level exceeds the upper level of the material with low permeability, and only a fraction flows down toward the depressions of the saline lakes. Most of the saline lakes in Nhecolândia show seasonal or permanent cyanobacterial blooms, depending on the intensity of rainfall during the wet season [41], [42], [43].

**Fig 3:**
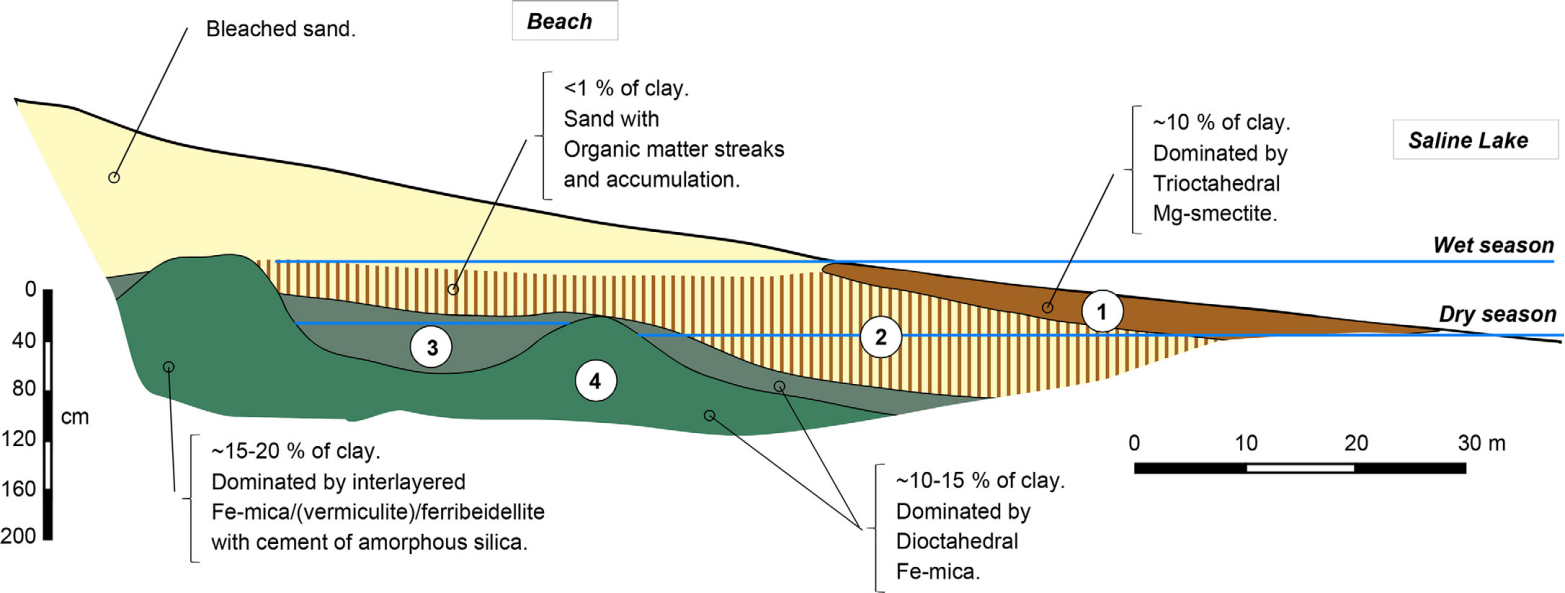
Standard soil layout and clay fraction distribution compiled from previous studies carried out around saline-alkaline lakes in Nhecolândia. See text for description of horizons 1 to 4.

The soils system around the saline-alkaline lake was studied by various authors in the western [44], northern [30] and southeastern [45], [32] part of Nhecolândia. All the studies revealed a similar soil organization that has been simplified on Fig 3, with mainly 4 contrasting horizons. Close to the lake, a grey-brown topsoil loamy sand horizon (1) is observed usually with numerous calcareous precipitations. The occurrence of this horizon is limited to the oscillation zone of the lakeshore between the wet and dry seasons. Its clay content is about 10%. Below, there is a light brown sandy material (2) coloured by vertically oriented organic matter streaks. The organic matter is also accumulated at the base [32], [44] or at the top [19] of this horizon [46]. The clay content in this sandy horizon is less than 1%. Subjacent to this horizon, there is a massive (single grain), greyish loamy sand material (horizon 3) with about 15% clay, and further below a loamy sand, olive to light olive-grey coloured horizon (4), with 15–20% clay, massive structure (coherent and cemented) and locally extremely firm consistency. The top of this horizon (4) is wavy with two main rises usually separated by 25 to 40 m.

The fine clay fraction in this alkaline soil system has been studied in detail by Furquim et al. [19], [30] [31] using X-ray diffraction (XRD), transmission electron microscopy-energy dispersive X-ray analysis (TEM/EDX), and inductively coupled plasma-mass spectrometry (ICPMS).

Smectites are concentrated at two places in the soil system [19]:

- In the rise of horizons 3 and 4, the furthest from the lake, the smectite is dioctahedral and was classified as ferribeidellite. According to these authors, the interstratification of this ferribeidellite with mica and vermiculite, and the Fe content suggest that the ferribeidellite originates from the transformation of previously neoformed Fe-illites (see below) and that vermiculite is an intermediate phase in this transformation.
- In horizon 1, smectites are dominantly Mg-rich trioctahedral smectite. They were identified as saponitic and stevensitic minerals resulting from chemical precipitation in the saline-alkaline soil solution [30].

The non-expanding 2:1 phyllosilicates are dioctahedral illite-micas, mainly concentrated in horizons 3 and 4, and interpreted as neoformed clay by Furquim et al. [31]. Among 8 crystals analysed by these authors, glauconite and Fe-illite are the dominant micas and have undergone degradation into vermiculite and ferribeidellite. The higher percentage of micas in the fine clay fraction reached up to 84% in horizon 4 close to the lake. Si-rich amorphous materials were associated with small crystallites in the mica-enriched horizon 4. Low-frequency electromagnetic induction surveys combined with direct observations (auger holes and excavated pits) revealed that mica-enriched horizons form a continuous ring around the saline lakes attesting to their authigenetic nature [40], [45], [32].

From this pedological framework, the major issue is to understand how Mg-smectite and Fe-mica can form in the same environment. The formation of trioctahedral Mg-smectites in the soil horizons at the immediate border of the lake necessarily indicates Si- and Mg-rich but Al- and Fe-depleted solutions. This contrasts with the presence of dioctahedral micas a few meters away, and for which the local source of Fe and Al and their transfer in the landscape appears as the main chemical constraint. No precursors that could explain the above chemical variations were identified, and both Mg-smectite and Fe-mica dominated horizons are in contact with the same watertable. Therefore, in the present study we address this paradoxical clay distribution by focusing on a representative saline lake. In a first step we verified through mineralogical, morphological and chemical characteristics the prevalence of this alkaline soil catena in the Nhecolândia region. Second, we focussed on the seasonal fluxes of water and chemical elements within the sand of horizon 2, between Mg-smectite-rich horizon 1, and Fe-mica-rich horizons 3 and 4.

## Material and Methods

The study was concentrated around a saline lake called “Salina Verde” (19°28’13”S and 56°3’22”W) located on a private land called “Centenario” farm (Fig 1), and was conducted with the permission of the owner. We acquired the field data only at unrestricted locations where no special permission was required and avoided the national park and other protected areas. The field studies did not involve endangered or protected species.

### Water level and electrical conductivity monitoring

The lake is supplied mainly by the local rainfall. Secondarily, during the flood peak, it receives fresh water by sub superficial flow from draining fields, locally called “Vazante” [47]. The upper level of the soil horizons with low permeability that isolate the saline lake was assessed by low frequency electromagnetic induction (EM31-MK2, Geonics, Ontario) according the relationship establish by Furian et al. [32]. The water level (h) and electrical conductivity (EC) were monitored from September 2010 to October 2011. Data were recorded automatically every 4 h using Schlumberger Mini- and CTD-Diver® pressure transducer combined with a Baro-Diver® to compensate for atmospheric pressure (both having 1.5 mm accuracy). The Mini-Diver was installed in a piezometer drilled in the draining fields at about 300 m from the lake. CTD-Divers were installed in the Salina Verde, and in a piezometer drilled on the beach between the two rises of horizon 4.

### Soil study

The first objective of the soil study was to verify that the site chosen at the border of Salina Verde was similar to that comprehensively described by Furquim et al. [30]. The soil was studied along a transect perpendicular to the lakeshore located so as to maximize the lateral changes in the soil, which is organized into rings surrounding the lakes [32]. Soil samples in contact with the collected solutions were taken during the high-water level period (June 2011). The samples were stored in an airtight container and completed (without air) with the circulating solution in order to prevent oxidation. The particle size was measured by the pipette method [48]. The soil samples were analysed by X-ray diffraction on oriented powder preparations dried at normal temperature and saturated with ethylene glycol. Two fractions were selected: the bulk material and the fine fraction isolated by sonication/elutriation. The spectra were obtained with angular (2θ) ranges from 2.3° to 34° and steps of 0.03°. The analyses were carried out using a G3000- INEL diffractometer (Cu cathode) equipped with a diode SiLi. Quantitative analyses were performed on 6 compacted fine clay (<0.2μm) patches using electronic microprobe SX50 fitted with wavelength dispersive spectrometers (WDS). Fine clay samples were also selected for Mössbauer spectra in order to characterize Fe location in the clay network. Spectra were collected on a constant-acceleration conventional spectrometer with a 0.30 GBq source of 57Co (Rh matrix) at 80K, using a MD306 Oxford cryostat. The thermal scanning was monitored by an Oxford ITC4 servo control device (0.1 K). The absorber was a sample of ca. 100 mg of powder that was enclosed in a 20 mm diameter cylindrical plastic sample holder, the size of which has been determined to optimize the absorption. Mössbauer’s parameters and their standard deviations of statistical origin were obtained by least-squares fitting to Lorentzian lines using Recoil software [49]. The isomer shift values are given with respect to metallic iron at room temperature. In addition, FTIR measurements were performed on oriented fine clay samples in the near infrared region over the wave number range of 4000–8000 cm^− 1^, using a Nicolet 6700 equipped with a Thermo-Scientific integrating sphere. White saline efflorescent crusts were collected during the dry season on the lakeshore and the bulk material was characterized by XRD and SEM using a Jeol JSM 6360LV Scanning Electron Microscope fitted with energy dispersive spectrometry (EDS).

### Water chemistry

Water samples were taken from auger holes in the water table during high and low water level in June 2011 and October 2013, respectively. The electrical conductivity, pH and redox potential were measured in the field. Water samples were centrifuged in the field immediately after the collection for 30 min at 6000 rpm (Relative Centrifuge Force = 3200 g). The supernatant solution was stored in dark and 4°C conditions, in HDPE bottles previously washed with dilute HCl. The centrifuged water samples were analysed before and after filtration at different threshold (0.45 μm, 30 KDa, 10 KDa and 3 KDa) using cellulose acetate filters and Amicon® ultrafiltration units, respectively. Membranes were previously rinsed with ultrapure water in order to avoid contamination. In a second step, the centrifuged solutions were diluted twice by addition of hydrogen peroxide (H_2_O_2_ 30%) in order to decompose the possible organic colloids and then filtered at 0.45 μm. The cations (Na, K, Ca, Mg, Si, Al, Fe) were analysed by high resolution ICP-MS (GET Laboratory, Toulouse, France) for samples collected in 2011 and by ICP-AES (Soil Science and NUPEGEL Laboratories, USP, Piracicaba, Brazil) for samples collected in 2013. Anions (Cl, SO_4_) were measured by HPLC, alkalinity by 0.1N HCl titration, and dissolved organic carbon (DOC) concentrations were determined with a Shimadzu TOC-5000A (CENA-USP, Piracicaba, Brazil). In order to compare the collected soil solutions with the regional chemistry presented by Furian et al. [32], the results were plotted into concentration diagrams based on their sodium amount.

### Additional data

Aerial pictures were selected from Google Earth™ at the end of a severe dry season (July 11^th^. 2006 and August 15^th^. 2010) in order to reveal the morphology of the bottom of these alkaline lakes.

## Results

### Soil mineralogy and distribution

**Fig 4:**
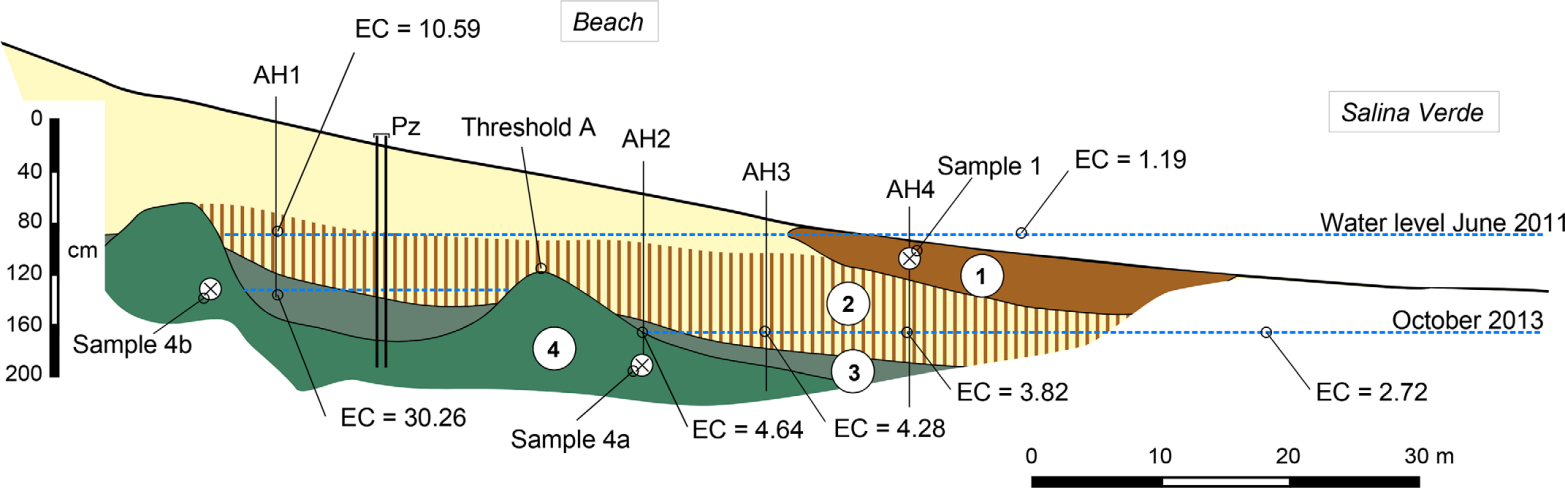
Cross section showing the soil layout along the transect. AH are auger holes for water collection, Electrical conductivity (EC) in mS cm^-1^, ⊗ are samples presented in Fig 5, Pz denotes the piezometer used for water level monitoring in the beach.

**Fig 5:**
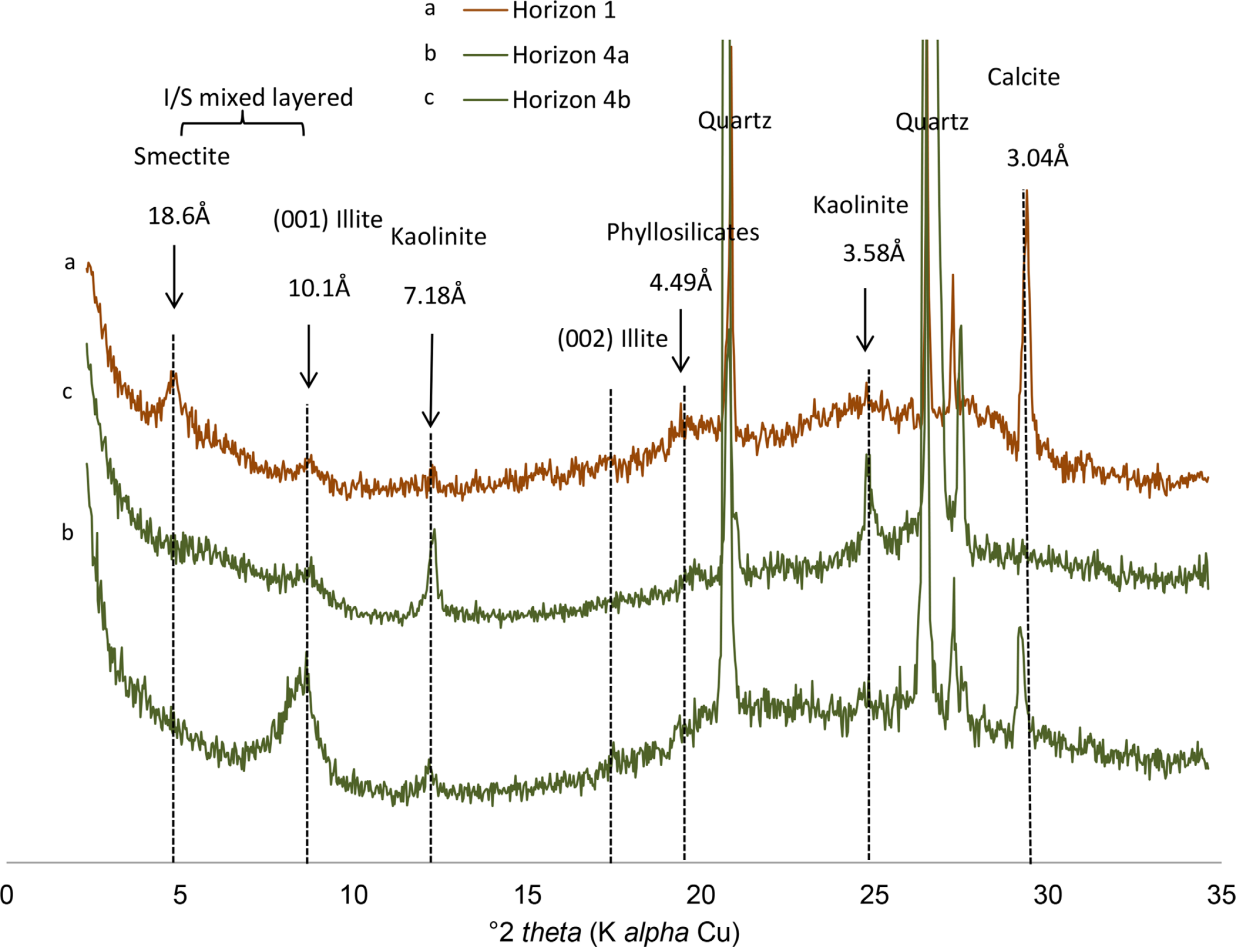
X-ray diffraction of glycoled-oriented clay along the transect showing smectite-dominant (1) and mica-dominant horizon (4).

**Fig 6:**
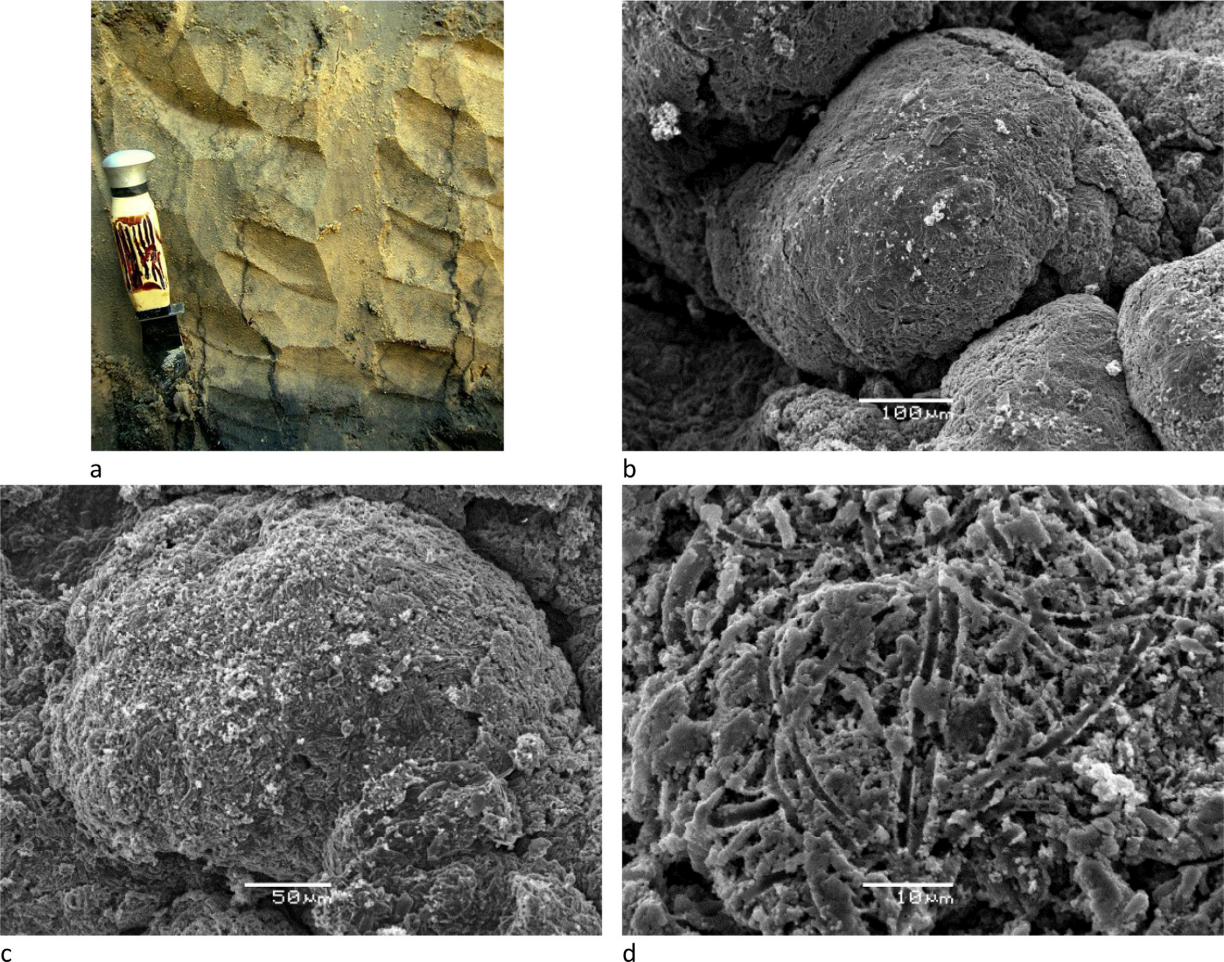
(a) Streaks of organic matter in horizon 2; (b, c and d) SEM pictures of the white efflorescent crust consisting of mats of amorphous material.

Four soil horizons have been identified and their distribution along the studied transect (Fig 4) appeared very similar to that summarized in Fig 3 from other studies in the region. In horizon 1, XRD patterns of bulk materiel (not shown) display a mixture dominated by quartz and smectite, secondarily mica, kaolinite and occurrence of calcite. Clay contents were about 10%, and oriented XRD spectra confirmed a predominance of smectite, secondarily of mica, I/S interlayered and kaolinite in the fine clay fraction (Fig 5). The microprobe analysis on 3 samples (clay patches) indicated that this fraction is composed of Si (57.1 to 62.7%), and secondary of Mg (13.9 to 17.7%), Al (4.2 to 4.6%), Ca (4.1 to 4.4%), K (3.1 to 3.3%), and Fe (2.9 to 3.7%). Such a mineralogical and chemical composition is similar to that reported from horizon 1 by Furquim et al. [19] using the same methods. Mg and Al are related to the phyllosilicates, K and Ca reflect the presence of illite and calcite, respectively. Below, the sandy material of horizon 2 was coloured by vertically oriented organic matter streaks (Fig 6A) and with organic matter accumulated at the base of the horizon. The clay content was less than 0.5% and XRD on the bulk material revealed only quartz. Subjacent to this horizon, greyish horizon 3 and olive horizon 4 were observed. The top of horizon 4 formed two main rises separated by approximately 25 m. The first rise was reached by the lake water level during the dry season, while the second one was achieved during the wet season suggesting that they arise from the present day hydrological regime of the lake. The clay contents were about 10 to 14%, in horizons 3 and 4, respectively, and the fine clay consisted almost exclusively of mica and kaolinite from the lake to the first rise of greenish horizon (horizon 4a on Fig 5), and of a mixture dominated by mica, secondarily smectite and kaolinite in the second one (horizon 4b on Fig 5), where a diffuse reflection on XRD spectra suggests the occurrence of interlayered mixed illite/smectite. In horizon 4 close to the lake, the microprobe analysis on 3 samples indicated Si (53.8 to 57.2%), Al (13.8 to 17.4%), Fe (10 to 11.1%), K (4.7 to 5.6%) and Mg (2.2 to 2.7%). The clay patches appear here more enriched in Al, Fe and K, with much less Mg, a chemical composition consistent with the predominance of mica when compared to horizon 1. Again, these chemical and mineralogical results are similar to those reported in Furquim et al. [30].

**Fig 7:**
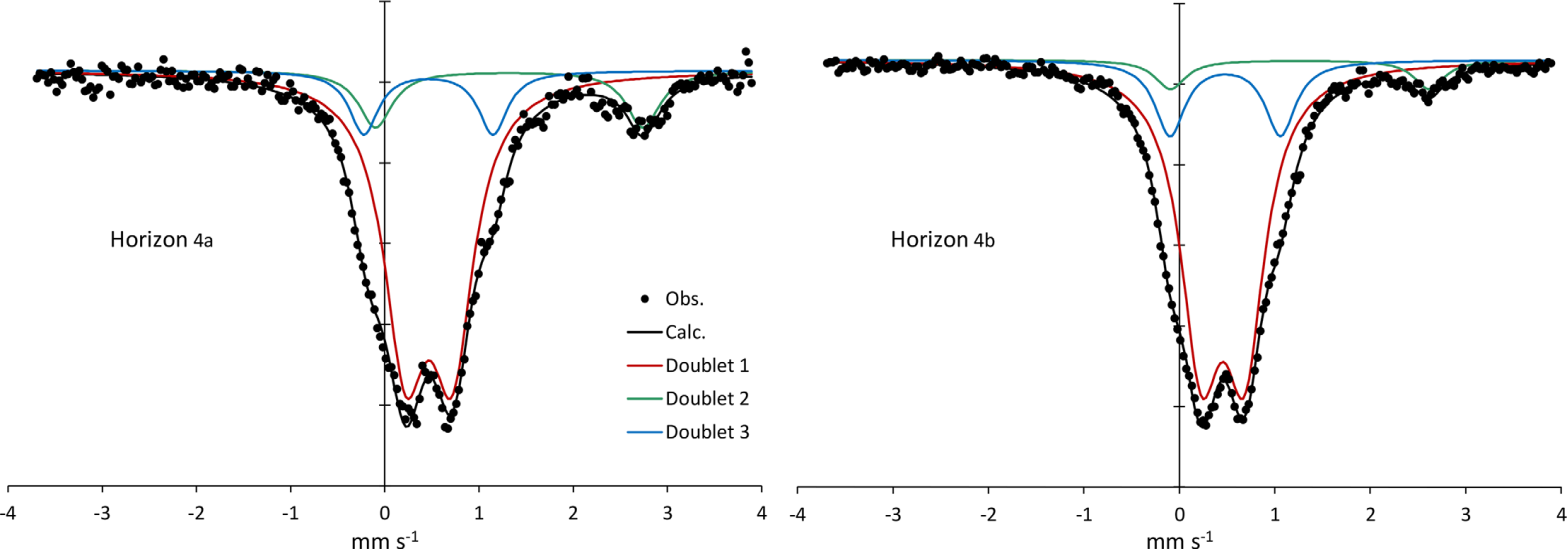
Mossbauer spectroscopy carried out on fine clay material of the greenish horizon 4 dominated by Fe-mica. The deconvolution into 3 doublets shows octahedral Fe^3+^ (doublets 1 and 3) and secondarily octahedral Fe^2+^ (doublet 2).

Mossbauer spectroscopy carried out on fine clay material (mainly mica) of the greenish horizon 4 showed complementary results (Fig 7). The best Lorentzian site analysis was obtained with three doublets of different intensity with distinct isomer shifts (δ) and quadrupole splitting (Δ) as presented in Table 1. The most visible doublets correspond to Fe^3+^ in the octahedral site (Doublet 1: δ ~ 0.47 mm/s and Δ = 0.5 mm/s) and Fe^2+^ in the octahedral site (Doublet 2: δ ~ 1.3 mm/s and Δ ~ 2.8 mm/s). The values of theses parameters are common in phyllosilicates and correspond to a Fe^2+^:Fe^3+^ ratio of 1:4 [50]. Doublet 3 (δ ~ 1.3 mm/s and Δ ~ 2.8 mm/s) had intermediate parameters and is more difficult to interpret. This is due to the difficulty to distinguish diamagnetic Fe(II) with low spin from Fe(III). According to some authors[51], [52] the intermediate parameters may correspond to tetrahedral Fe^3+^. But other studies privilege octahedral Fe^2+^ [53]. In any case, we consider that the Mossbauer data, even if some interpretations may be controversial, do not clearly contradict the assumption of octahedral Fe^3+^.

**Table 1:** Mössbauer spectrometry characteristics of fine clay mica from horizon 4.

	Chemical shift δ	Quadrupole Splitting Δ	Amplitude	width at half size w+	Site Populations
	(mm s^-1^)	(mm s^-1^)	(counts mm s^-1^)	(mm s^-1^)	(%)
**Sample 4a**					
** Doublet 1**	0.4680	0.499	56500	0.275	75.2
** Doublet 2**	1.322	2.841	9600	0.216	12.7
** Doublet 3**	0.463	1.371	9100	0.185	12.1
**Sample 4b**					
** Doublet 1**	0.4558	0.457	108400	0.256	77.7
** Doublet 2**	1.26	2.7	8800	0.195	6.3
** Doublet 3**	0.48	1.16	22200	0.191	15.9

**Fig 8:**
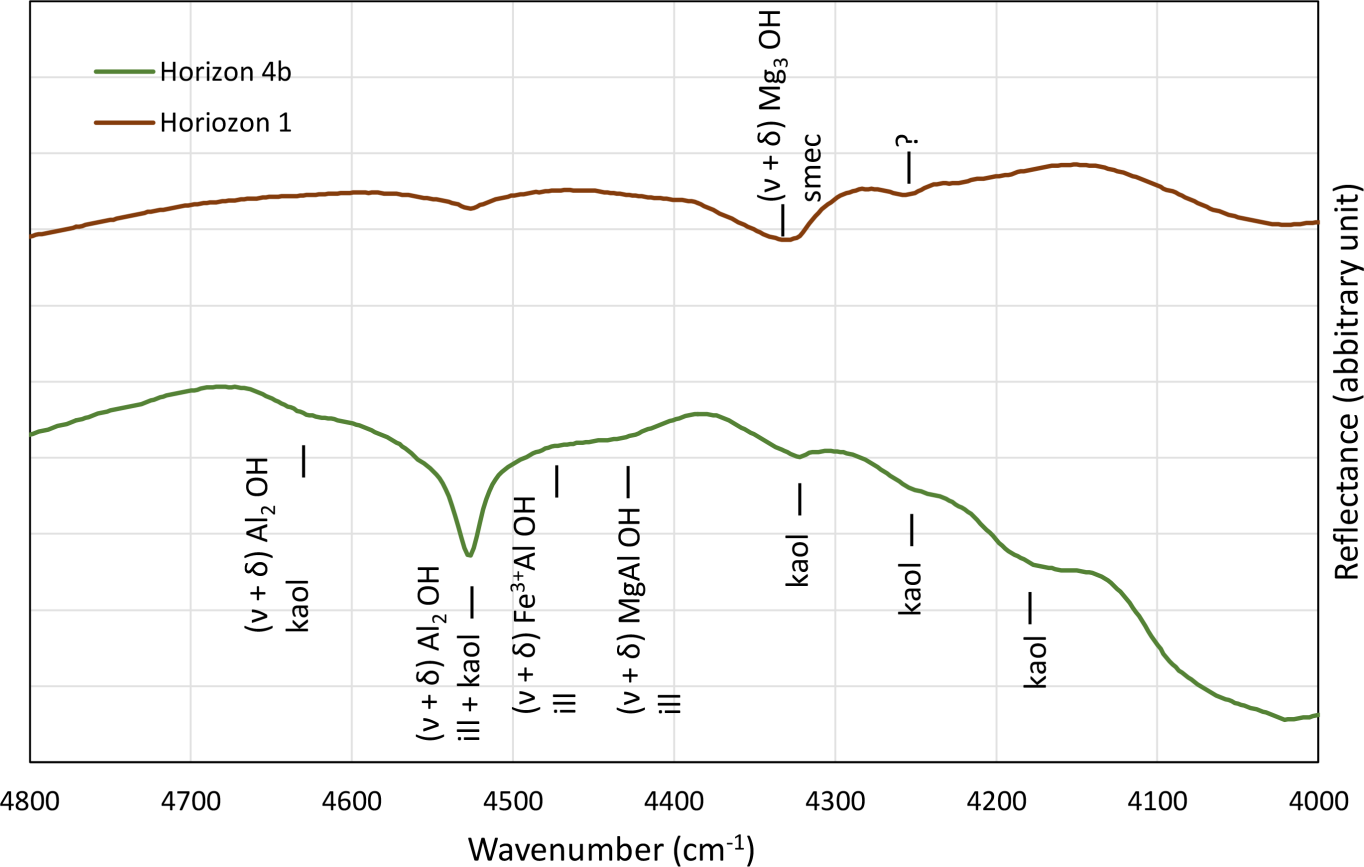
Near infrared reflectance on fine clay material of horizon 1 and 4. V and δ denote stretching and bending, respectively.

The presence of kaolinite in the samples masked the information regarding other clay minerals on FTIR spectrum. However, Fig 8 showed the presence of dioctahedral minerals, mainly in horizon 4b, with shoulders at 4430 cm^-1^ and 4463 cm^-1^, related to AlMg–OH and AlFe^3+^–OH bonds, respectively, which cannot be attributed to kaolinite. On the other hand, the reflectance at 4330 cm^-1^ denoted the presence of trioctahedral minerals in horizon 1 related to Mg3–OH bond.

The white efflorescence consisted of clusters of approximately 500 μm composed of mats of tubules of amorphous silica (Fig 6B, 6C and 6D). The X-ray diffraction pattern revealed a large dome without clearly crystallized material, with the exception of quartz and calcite.

### Hydrological functioning

**Fig 9:**
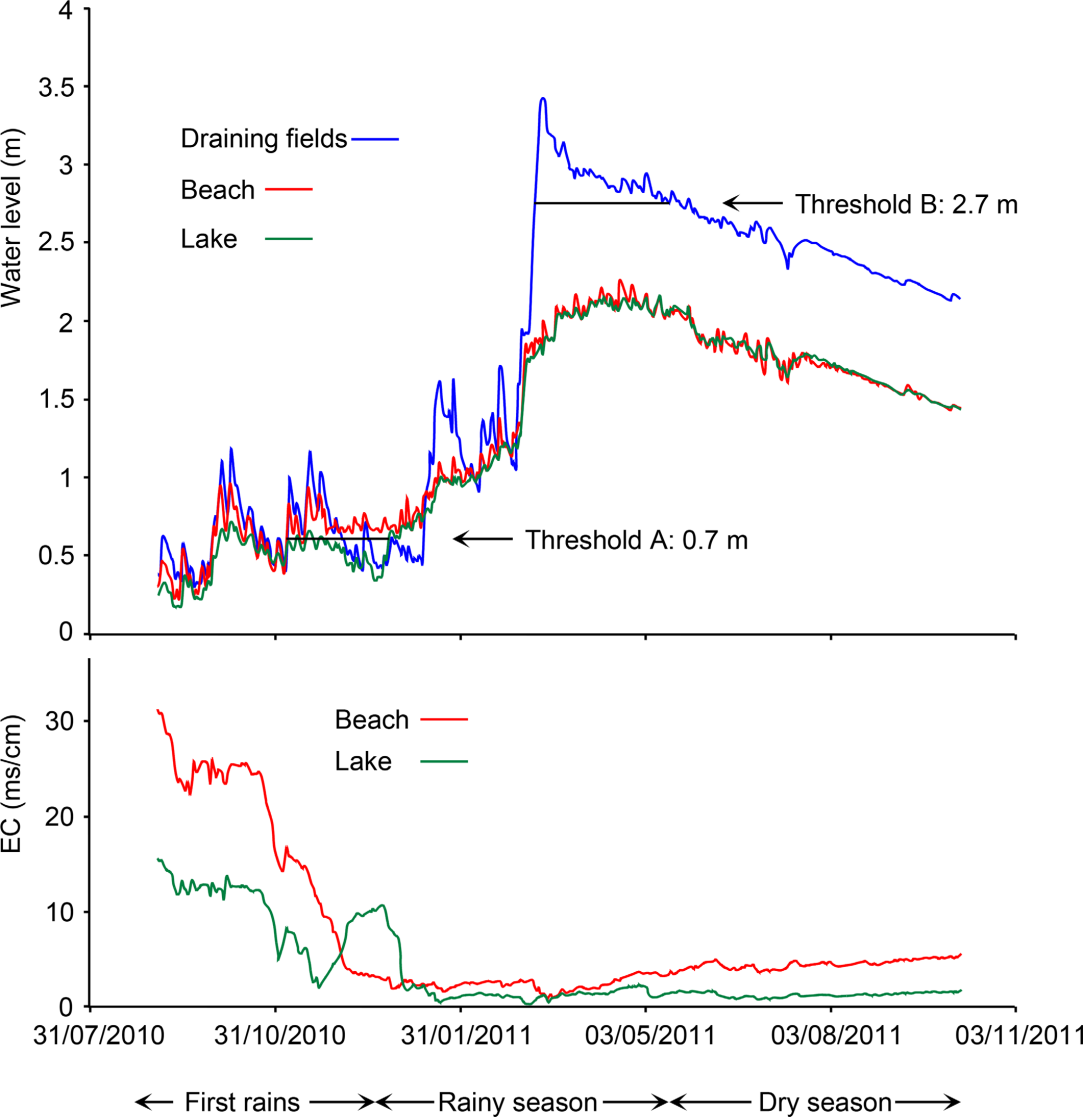
Water level monitoring of the fresh watertable of the draining fields, the saline beach groundwater and Salina Verde. Level zero corresponds to the bottom of the lake.

Fig 9 shows the water level and EC monitoring during 2010–2011, with low water levels and high EC values in early September 2010 (15 and 30 mS cm^-1^ in the lake and in the beach groundwater, respectively). The 0 m level in Fig 9 corresponds to the deepest point of the lake. Electromagnetic induction survey combined with auger observations allowed for identifying two rises in the sandy loam horizons with low permeability that act as threshold in the hydrological functioning of the draining fields-saline lake system. The first one (A), at about 0.7 m high, was the first rise of horizon 4, the closest to the lake (Fig 4). The second one (B) at 2.7 m was located in the forest at about 100 m from the lake. The influence of this second threshold in the seasonal hydrology was already described from other lakes of Nhecolândia [54], [40] and Furian et al. [32] have shown that such a functioning can be generalized to the region. The water levels oscillated under the influence of the first rains of the season, and EC decreased gradually in both, the lake and the beach groundwater. From early November 2010, the beach groundwater level stabilized at a level slightly higher than the threshold (A), and it was associated with a steeper decrease in the EC. Under these conditions, the beach groundwater can flow to the lake. The EC then increased sharply in the lake, up to about 10 mS cm^-1^, and this value was maintained for about 1 month. During the rainy season, the lake level gradually increased, exceeded threshold A, and from there, both lake and beach groundwater levels were similar. From mid-March to mid-May, the water level in the draining fields exceeded threshold (B). Freshwater can then flow from the draining fields towards the lake, which was reflected in a slight EC decrease, especially visible in the beach groundwater. Then, the water level in the draining fields repassed below threshold (B) and stopped supplying the lake. Levels then decreased under the influence of evaporation. Although the water levels in both the lake and the beach were similar, EC increased more rapidly in the beach groundwater. It is in these conditions that the water samples were collected during the dry season 2013.

### Inorganic and organic chemistry

**Fig 10:**
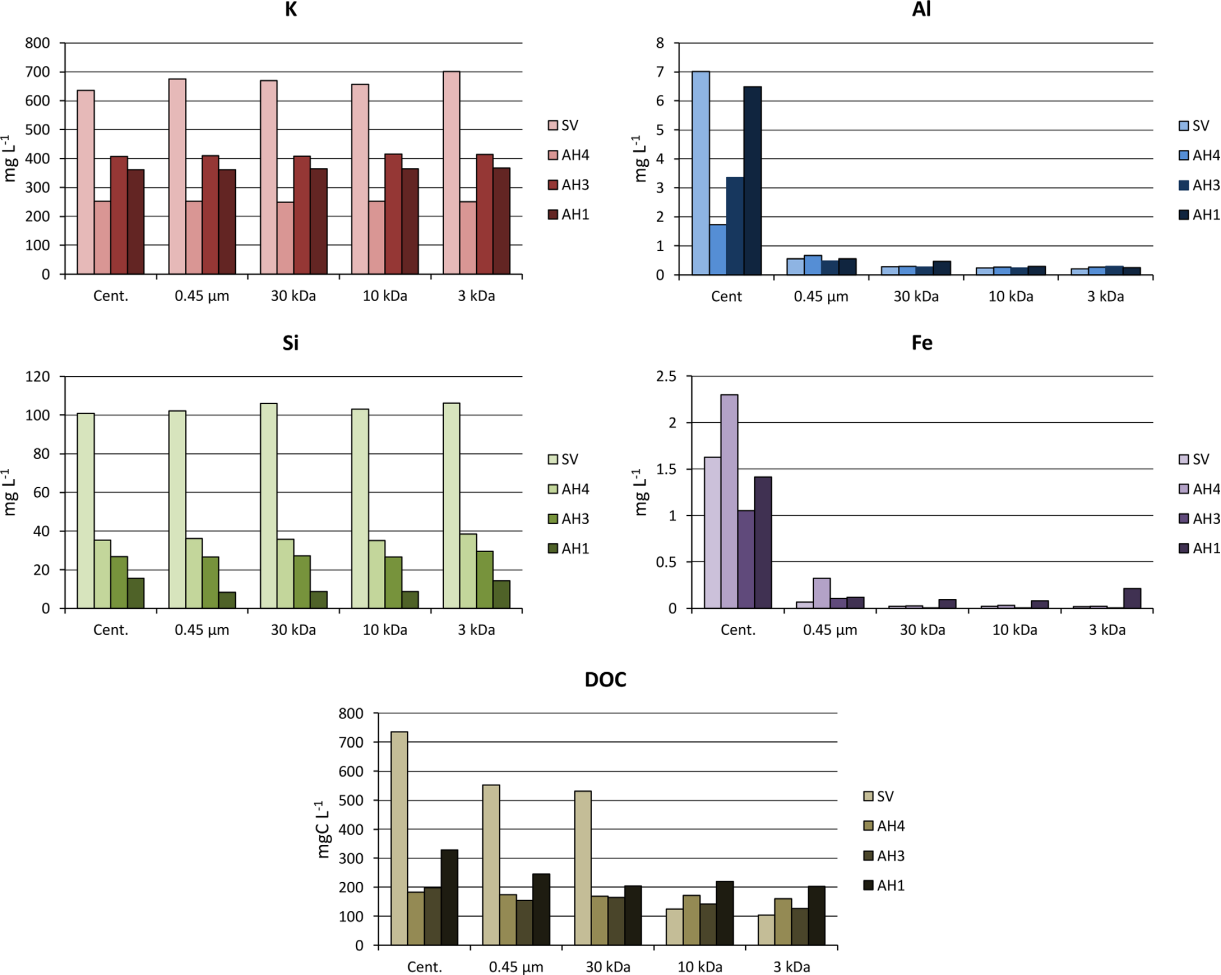
Si, K, Fe, Al and DOC contents in solutions after centrifugation and subsequent filtration at 0.45 μm, 30 kDa, 10 kDa and 3 kDa. SV denotes Salina Verde and AH are auger holes shown along cross section in Fig 4.

**Fig 11:**
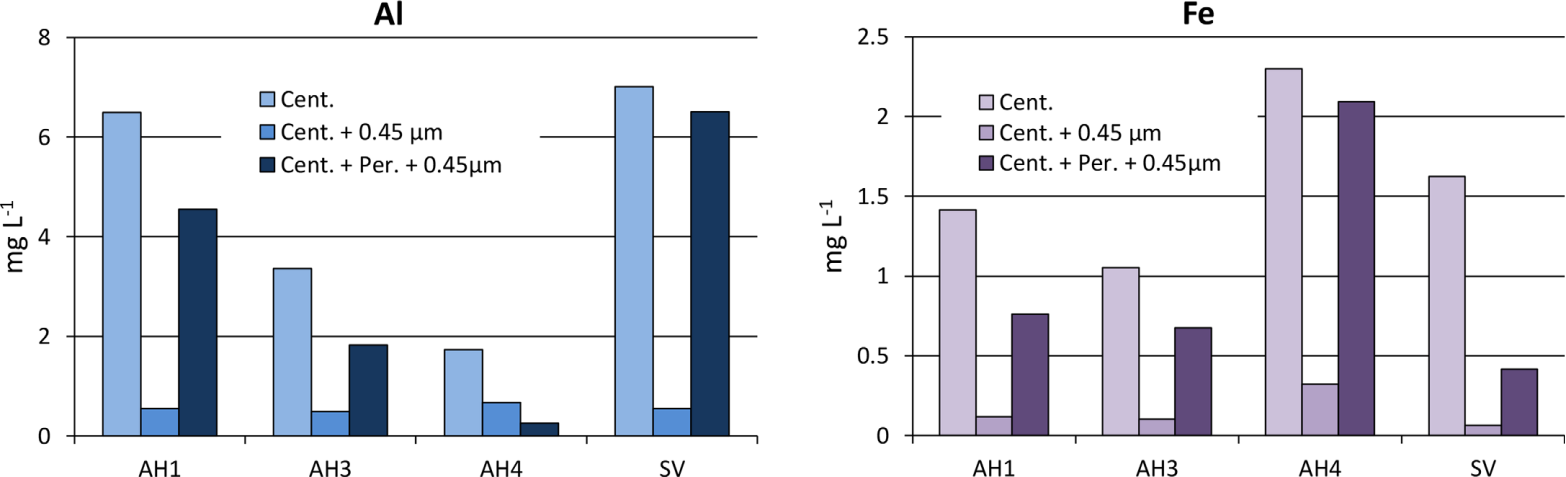
Fe and Al contents in centrifuged solutions, centrifuged and filtered solutions, and after hydrogen peroxide treatment and subsequent filtration at 0.45 μm. The peroxide treatment tends to reduce the proportion of Fe and Al retained by filtration.

The soil pH of all the horizons described below was alkaline, with values ranging from 9.2 to 10.9. Redox potentials were of about –0.36 V and +0.3 V in the lake sediments and water, respectively, and ranged from -0.3 to -0.1 V in the beach water table. EC values reported in Fig 4 confirmed that the water electrical conductivity increased steadily from the lake to the beach groundwater by a factor of about 10, for example from 1.19 to 10.59 mS cm^-1^ in June 2011. The maximum EC was observed between the two rises of the greenish horizon at the end of the dry season (October 2013). The chemistry of major elements in the lakes and water table (S1 Table) was consistent with the regional trends drawn with solid line on Fig 2 from the data reported by Furian et al. [32]. The amounts of Cl^-^ and SO_4_^2-^ were fairly low but increased in proportion to Na^+^. Ca^2+^ and Mg^2+^ contents decreased, while K^+^ and the carbonate alkalinity increased but less rapidly than Na^+^. DOC value in Salina Verde was very high up to 738 mg L^-1^ and high values ranging from 175 to 329 mg L^-1^ were also observed in the saline water table. These values are of the same order of magnitude as that reported by Mariot et al. [46] in other sites of Nhecolândia. The chemical composition of the solutions after centrifugation and filtration are shown in Fig 10. Fe and Al values were high in the centrifuged solution, showing a trend to increase with increasing DOC values. The different filtrations had no influence on Si and K contents. In contrast a noticeable decrease of about 90% of Fe and Al contents was observed from the first cutoff threshold of 0.45μm. Half of the remaining Fe and Al disappeared with further filtration mainly at a 30KD threshold. After hydrogen peroxide treatment and when compared with centrifuged solution, 15 to 92% of initial iron and aluminium contents are recovered and are no longer retained by the 0.45μm filtration (Fig 11 and S2 Table).

## Discussion

### Regional processes

The study site and the processes occurring in the soil system at the border of the saline-alkaline lakes appear representative of the Nhecolândia region. It is the first aspect of this discussion, which can be addressed through the soil morphology, the distribution, mineralogy and chemistry of the clay fraction, and the soil solution chemistry. The morphological similarity between the soil sequence at the Salina Verde and that summarized from previous studies (Figs 4 and 3, respectively) in various places of Nhecolândia indicates that the same pedogenic processes are occurring, leading to a standard alkaline soil system throughout the region. The similarity is reinforced by the distribution of phyllosilicates. The soil layout stresses the opposition between a topsoil horizon close to the lake mainly with smectite, and a deep greenish horizon dominated by mica. For the mica, the low intensity of the (002)/(001) reflection peak ratio (<0.1, Moore and Reynolds, 1989) and the good quality of the Mössbauer spectra suggest the presence of Fe-rich mica. The chemical composition assessed from clay patches is close to that given by Furquim et al. [19], [30] for Mg-smectite (stevensite and saponite) and Fe-rich mica, respectively. The reflectance in the near infrared is also in agreement with trioctahedral minerals in topsoil horizon (1) and dioctahedral minerals in horizon (4). Therefore, we assume that we are in the same pedological context as that comprehensively described by these authors at about 90 km from our study site. Finally, the major elements chemistry (Fig 2) along the studied sequence being similar to that observed at the regional level by Furian et al. [32], the associated processes are also representative of the region.

### Hydrochemical functioning

Along the studied catena, both the Mg-smectite and Fe-mica dominating horizons are separated by the sandy horizon (2) with many organic streaks, emphasizing the dynamics of the organic matter in the system, which is confirmed by the high DOC values up to 738 mg L^-1^ in the lake water and about 200–300 mg L^-1^ in the beach water table. The water trapped between two rises of greenish horizon (4) with low permeability is quickly isolated from the lake water table during the dry season. Because of its low volume, it reaches high conductivity under the influence of evaporation. After the first rains, this groundwater reservoir is supplied with freshwater probably arising from the rainfall in the upper part of the beach (upslope threshold A), and the alkaline brine is pushed down to the lake by piston effect, provoking the sharp increase in lake water EC. After April 2011, the increasing EC values show that solutions concentrate from the lake towards the beach that promotes evaporation by wicking. This water transfer prevails during several month of the dry season, and favors the transfer of solutes towards both, horizon (1), in particular Si and Mg to form Mg-smectite, and horizon 4, including Si, K, Fe and Al necessary to form mica.

The results of the successive filtrations confirm that the transfer of silica (~100 mg L^-1^) and potassium (~700 mg L^-1^) is achieved as dissolved form through horizon (2). Although K contents increase with increasing evaporation, a loss of about 85% of K occurs when compared with Na equivalent enrichment (Fig 2) in the soil system that surrounds the lake, and it can be attributed to K incorporation in the layers during the formation of Fe-mica. Si contents are mainly controlled by the alkaline pH that provokes the dissolution of the orthosilicic acid. The white efflorescent crusts confirm the dynamics of silica along the alkaline catena. Although the alkaline brines seems to be at the equilibrium with magadiite [40], this Na-silicate was not identified on XRD spectrum. Fe and Al have low solubility and mobility in non-acidic natural environments. Here, the low redox potentials measured could favor Fe reduction but even the soluble form Fe^2+^ is not stable under high pH conditions. Fig 10 shows that Fe and Al contents strongly depend on the filtration that decreased their amounts by a factor of 10 from the 0.45-μm cut-off threshold. This observation suggests a colloidal control for Fe and Al transportation that is significant for the coarser fraction only. The treatment with hydrogen peroxide damages these molecules confirming their organic nature, and releases a large fraction (15 to 92%) of these metal ions (Fig 11). Two factors may explain that the amounts of Fe and Al recovered after peroxide treatment and filtration (treatment Cent. + Per. + 0.45μm) remained below the levels obtained in the centrifuged solutions (treatment Cent.). The first factor is an incomplete destruction of the organic matter, and the second is the rapid formation of iron and aluminium (oxy)hydroxides of size greater than 0.45μm just after the organic ligands digestion by peroxide.

### Mineralogy distribution

**Fig 12:**
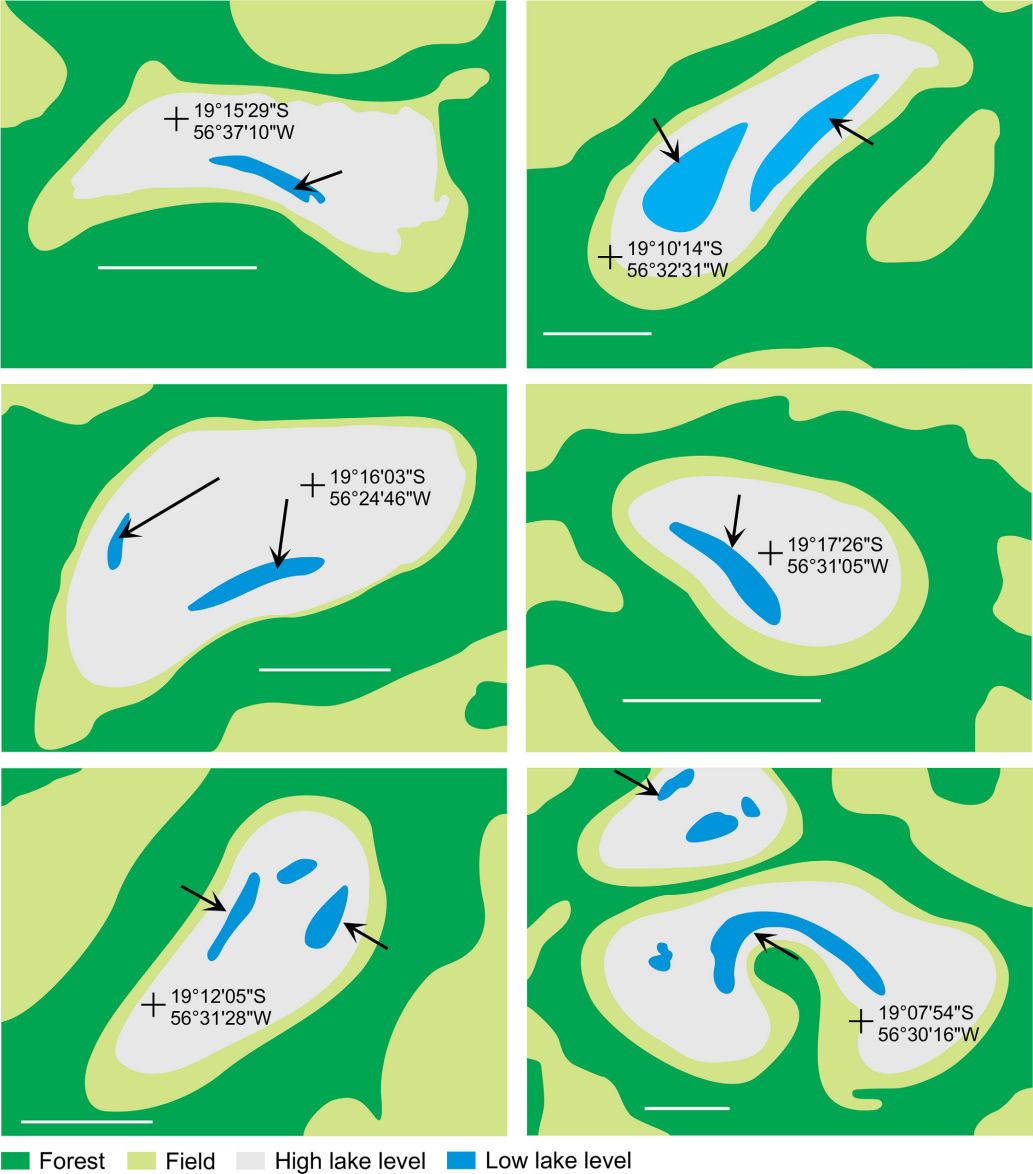
Examples of alkaline lakes in the Nhecolândia showing elongated deep borders (arrows) suggesting that the chemical withdrawal-aggradation process impacts the lake depression. Bar = 200 m. Original pictures are available at Google Earth^TM^ imagery.

The chemical and structural changes of the clay fraction in this catena are obviously constrained by the availability of the constituents. Three origins of the elements should be taken into account: mineral precursors, the aqueous species and a colloidal source. Although usually not considered, the latter is of particular interest here, since it can compensate the low aqueous Al and Fe concentrations. Clay diagenesis is generally the result of the slow and continuous transformation of a precursor: the conversion of smectite into illite for example, proceeding by successive steps of interlayered assemblage. In the present case, the mica structure identified in horizon (3) and (4) was interpreted as resulting from neoformation by Furquim et al. [30]. The absence of a magnetic component in the Mössbauer spectra suggests that all of the iron is incorporated within the phyllosilicate structure. Fe is mainly Fe^3+^, secondarily Fe^2+^ in octahedral layers. Therefore, this Fe-mica is predominantly dioctahedral mica, which is also in agreement with FTIR analyses. The interlayered I/S detected here by XRD is rather the result of the mica degradation by a posterior process [19]. The immediate question for a direct precipitation of a 2:1 dioctahedral phyllosilicate is the source of the less soluble constituents Fe and Al. The rises of horizon (4) suggest that the precipitation of mica occurred in a previously sandy material similar to that observed in horizon (2). A local precursor can be consequently discarded. The presence of saponitic and stevensitic smectites in horizon (1) is clearly another occurrence of neoformed clay by direct precipitation from the soil solution [19]. However, the absence or the low Al and Fe contents in these clays makes it impossible as a source of Fe and Al for the illite-mica. By contrast, the high concentration of organic molecules, in particular possible organo-metallic complexes, constitutes a local source of Al and Fe when it decomposes in the alkali horizon. The mineralization of the organic matter, when it occurs, is likely a faster reaction than the silicate dissolution and may drive a high aqueous iron and aluminium activity. The high DOC and total Al and Fe contents in the lake water suggest that the primary source of Al and Fe is likely from the detrital silicates below the lake sediments. In the long term, the dissolution of primary minerals (quartz and other silicates) under alkaline conditions and the lateral transfer of the released elements, in dissolved form or bond with organic matter, may impact the topography of the landscape, including the depression of the lake. This is consistent with Fig 12 drawn from aerial pictures that reveal that the deepest parts of the lakes are rarely located at the centre, but usually consist of elongated depressions along the beach. Although no picture was available for our study site, a similar feature exists in Salina Verde. This seems consistent with the fact that the internal and external borders of the alkaline lakes are the most active portions of the chemical withdrawal-aggradation process. The substantial mass transfer between the lake sediments and horizons 3 and 4 in the beach can be observed today. It contrasts with the slow kinetics of the smectite-to-illite conversion as observed in sedimentary basins [55], [56], [6], [57], even if alkaline solutions promote mineralogical changes in the 2:1 layer of smectites towards mica structures [58]. The incorporation of iron in the structure is not typical and may perhaps be considered as a possible key of low-temperature mica crystallization. In soils and more generally in sedimentary basin, the availability of aluminium and potassium is a constraint for clay diagenesis [59].

### An integrated model

From the soil mineralogy, water chemistry and the lake Hydrology, we propose a probable functioning of this alkaline soil system. The driving force behind the massive transfer of Si, K, Fe and Al from the lake to the beach is the evaporation process. The functioning of the system requires high DOC concentrations, and therefore alkaline solutions with pH close to 10 that favor high contents of soluble organic matter. These are guaranteed by water evaporation in the chemical context of positive calcite residual alkalinity, together with the singular hydrology of these lakes characterized by a negative water balance [32]. The permanent transfer of these elements also requires significant and regular input of organic matter, which is provided by permanent or seasonal blooms of nitrogen-fixing cyanobacteria in the lake [42], [43]. Finally, it requires the release of Fe and Al at a short distance. The produced algal organic matter, substantially cellulosic, is easily mineralized, and may allow the massive release of iron and aluminium in a short distance. At this stage of the study, it is not possible to confirm if the colloids of metal-organic molecules are complexed or simply adsorbed on the surface. Fe and Al could also be part of the chemical formula of the organic compounds as *Anabaena* communities are known to accumulate metal within cell walls and thylakoids membranes [60], [61]. This aspect should be confirmed by further studies on organic matter characterization, binding affinity for Fe and Al, and mineralization rates along the studied soil transects.

### Similarities with other alkaline environments

On the one hand, although the conditions of formation of trioctahedral Mg-smectites are today widely accepted and used for the reconstruction of alkaline paleo-environments, many authors have also mentioned the occurrence of micas (non-expanding clay minerals with 1 nm basal spacing), and usually fine-grained Fe-illites, as a dominant clay mineral in alkaline soil systems [25], [62], [18], [63], [64]. In addition, many of these authors recognized that the origin of such illites is uncertain, but they are usually considered as probably detrital clay minerals, due to the lack of geochemical arguments to explain the co-existence of Mg-smectite and Fe-illite, or any systematic trends in the relative proportions of illite and smectite from different parts of the studied soil systems that could provide clues as to the origin of fine-grained illites through transformation process [64], [65].

On the other hand, the geochemical control of potassium in saline alkaline waters and soil solutions has been frequently described. In the Lake Chad basin, Maglione [66] described a pronounced loss of K with respect to chloride for the beach and island lake groundwaters during the earlier stages of evaporative concentration. Around Lake Magadi, Eugster [10] points out that K is lost even more extensively than alkalinity, and the loss corresponds to about 70% of the total potassium compared to sodium equivalent enrichment, even for moderate sodium concentration (Na ~ 300 mmolc). The author attributes the K loss to probable exchange on reactive surfaces that could be volcanic glasses or silicate gels. Similar proportions in K loss have been described in alkaline soil solutions beneath the islands of the Okavango delta [67]. These authors mentioned indications that K is being removed from the groundwater to form K-minerals in the deeper soils under the central zone of the island. In all these studies, K follows a sigmoid behavior in concentration diagrams similar to that observed in Fig 2 for the Nhecolândia region. This sigmoid trend has already been mentioned by Eugster and Jones [68], but never reproduced using thermodynamic models in supergene conditions.

## Conclusion

Alkaline environments in Nhecolândia provide an excellent modern system to understand clay origin and occurrence in alkaline soil systems and more generally in alkaline basins. Above described observations provide evidence that the soil processes on the shoreline of the alkaline lakes are controlled by the water evaporation flowing towards the beach by wicking. The associated secondary minerals are primarily Mg-calcite, trioctahedral Mg-smectites and dioctahedral Fe-micas. These precipitations generate a standard soil system characterized by the coexistence of, on the one hand, clays that form in Fe- and Al-depleted environments, and on the other hand, clays that requires Fe- and Al-rich solutions for their formation. Our results suggest that Fe and Al, required for the formation of mica, are bonded with the organic matter that renders them non-available at the immediate border of the lake. Mainly trioctahedral Mg-smectite can form. On the other hand, the dissolved organic matter makes these elements movable, and in concentrations that are at least 10 times higher than what was previously described from 0.45 μm filtered soil solutions. A few meters away, Al and Fe are released probably because of the mineralization of the organic matter, and then Fe-mica can form. The co-existence of trioctahedral Mg-smectite and dioctahedral Fe-mica should therefore be regarded as a standard occurrence in alkaline soil systems with organic rich waters.

## Supporting Information

S1 Table Lake and beach water table chemical data. (XLS)

S2 Table Water chemistry after treatments (centrifugation, filtration, peroxide addition). (XLS)
